# Use of action planning to increase provision of smoking cessation care by general practitioners: role of plan specificity and enactment

**DOI:** 10.1186/s13012-014-0180-2

**Published:** 2014-12-30

**Authors:** Marjolein EA Verbiest, Justin Presseau, Niels H Chavannes, Margreet Scharloo, Ad A Kaptein, Willem JJ Assendelft, Mathilde R Crone

**Affiliations:** Department of Public Health and Primary Care, Leiden University Medical Centre, Leiden, 2300 RC The Netherlands; Institute of Health and Society, Newcastle University, Baddiley-Clark Building, Richardson Road, Newcastle upon Tyne, NE2 4AX UK; Department of Medical Psychology, Leiden University Medical Centre, Leiden, 2300 RC The Netherlands; Department of Primary and Community Care, Radboud University Nijmegen Medical Centre, Nijmegen, 6500 HB The Netherlands

**Keywords:** General practise, Smoking cessation care, Implementation, Action planning

## Abstract

**Background:**

Strategies are needed to help general practitioners (GPs) promote smoking cessation as recommended by guidelines. This study examines whether the quality of action planning among GPs improves their provision of smoking cessation care.

**Methods:**

The effectiveness of a 1-h training programme was examined in a cluster randomised controlled trial in which 49 GPs participated. GPs who followed the training (intervention group; *n* = 25) formulated action plans related to i) enquiring about smoking, ii) advising to quit smoking, and iii) arranging follow-up for smokers motivated to quit. GPs also formulated a coping plan for encountering smokers not motivated to quit. The quality of these plans (plan specificity) was rated and, 6 weeks after the training, GPs reported on the performance of these plans (plan enactment). Multilevel regression analyses were used to examine the effects of plan specificity and plan enactment on patient-reported smoking cessation activities of the GPs in the intervention group (*n* = 1,632 patients) compared with the control group (*n* = 1,769 patients).

**Results:**

Compared to the control group, GPs who formulated a highly specific action plan during the training asked their patients about smoking more often after the training compared to prior to the training (OR 2.11, 95% CI 1.51–2.95). GPs were most likely to have asked patients about smoking after the training compared to prior to the training when they had enacted a highly specific formulated action plan (OR 3.08, 95% CI 2.04–4.64). The effects of GP plan specificity and plan enactment on asking patient about smoking were most prominent among GPs who, at baseline, intended to provide smoking cessation care.

**Conclusions:**

A highly specific action plan formulated by a GP on when, how, and by whom patients will be asked about smoking had a positive effect on GPs’ asking patients about smoking, especially when these professionals also reported to have enacted this plan. This effect was most prominent among GPs who intended to provide smoking cessation care prior to the intervention. Training in devising personalised coping plans is recommended to further increase GPs’ provision of advice to quit smoking and arranging follow-up support to quit smoking.

## Background

Current guidelines recommend that general practitioners (GPs) routinely ask patients about smoking, advise them to quit, assess their motivation to quit, assist them with quitting, and arrange follow-up quit smoking support (the 5-A Model) [1],[2]. However, GPs report difficulties when translating these guidelines into practise [3]-[7], resulting in a substantial gap between evidence and practise. A study in Dutch general practise showed that 79% of all smokers and 40% of smokers who discussed smoking with their GP did not receive stop-smoking advice [8]. The development of strategies that facilitate the implementation of guideline-recommended smoking cessation care may result in more patients being advised to quit and being provided with evidence-based quit-smoking support and, ultimately, giving up smoking [9]-[11].

Strategies to facilitate the implementation of evidence-based clinical guidelines often focus on influencing the behaviour of the health-care professionals [12]-[15]. Efforts to change the clinical behaviour of health-care professionals often involve didactic modes of delivery aimed at educating these professionals [13]-[15]. However, this approach implies a lack of knowledge and assumes that additional knowledge will change the behaviour of health-care providers, neither of which may necessarily be true. In fact, enhancing knowledge alone may not be the best, or even an adequate strategy, to influence the clinical behaviour of health-care professionals [16]. Similarly, the motivation and/or the beliefs of GPs to routinely adopt evidence-based guidelines are not always a reliable predictor of the routine implementation of these guidelines [17].

Psychological theories may provide a basis for identifying the predictors of GP behaviour and of behaviour change [16]. Clinical practise is a form of human behaviour that is sensitive to theory-based strategies that have proven effective in patient samples [18]-[22]. However, a systematic review showed that only a minority of the 235 interventions that previously aimed to facilitate guideline implementation by health-care professionals actually used those theory-based strategies [12].

One of the well-established theory-based strategies (albeit in other populations) is the self-formation of ‘conditional plans’, such as action plans and coping plans [23],[24]. Action plans link a situational cue to behaviour in order to promote behaviour change and habit formation, e.g. ‘*if X occurs (if the patient visits me because of a cough more than three times a year), then I will do Y (I will advise the patient to quit smoking)*’. Coping plans anticipate potential barriers to behaviour change which impede action plans from working. Such plans aim to bridge the gap between the individual’s intention to perform the behaviour and the actual performance of that behaviour [25],[26].

The mechanisms that underlie the effectiveness of action and coping plans involve a heightened accuracy and speed of detecting the contextual cue for performing the intended behaviour [27]-[30]. Plans that are more specific are suggested to result in a greater improvement of the intended behaviour compared to incomplete or vague plans [31],[32]. In addition, studies have shown that individuals who act according to their formulated action plans (i.e. plan enactment) are more likely to benefit from their plans, e.g. enacting an action plan to remove all tobacco products results in a higher likelihood to actually quit smoking [33],[34]. The effects of plan specificity and enactment on behaviour are strongest among those individuals who are the most motivated to change the intended behaviour [31]-[33],[35].

It has been shown that planning predicts the clinical behaviour of GPs in various clinical conditions [36]-[38]. Moreover, an intervention study showed that incorporating planning in postgraduate education increased the use of a practitioner-guided procedure among mental health professionals [35]. However, to our knowledge, no studies have examined whether planning improves the provision of evidence-based smoking cessation care by GPs.

The present study incorporates action planning within a training session for GPs, aimed at increasing their provision of smoking cessation tasks as recommended in clinical guidelines, including asking patients about smoking, advising them to quit, and arranging follow-up quit smoking support for smokers who are motivated to quit. Because GPs often indicate that patients’ lack of motivation to quit may act as a barrier to the provision of guideline-recommended smoking cessation care [5],[39],[40], GPs also formulated a coping plan to address this potential barrier.

Based on the reported positive effects of action planning in patient samples [41]-[43], we hypothesised that GP action planning would improve the performance of these smoking cessation tasks. Secondly, we hypothesised that formulating a coping plan for smokers who are not motivated to quit provided GPs with a solution for this type of barrier, thereby increasing the provision of smoking cessation care for this group [38],[44]-[48]. Since the present GP training includes multiple behaviour change strategies, we also examined the nature of action planning including plan specificity and plan enactment. In line with the previous findings on plan specificity and self-reported plan enactment [31]-[35], we hypothesised that GPs who formulated a highly specific plan and reported a high level of plan enactment would be more likely to provide smoking cessation care post-training. Finally, we hypothesised that these effects would be most evident among GPs with high intention to routinely implement smoking cessation care prior to the training.

## Methods

### Design and intervention

The present paper reports the results of a two-group cluster randomised controlled trial in general practise. GPs were randomly assigned to either the intervention or control condition. The intervention entailed a 1-h individual training session for GPs in the delivery of smoking cessation care. The training was based on behaviour change techniques related to methods that underlie the current Dutch guidelines for treating tobacco addiction (the 5-A model [2],[49]): 1) GPs’ implementation barriers were identified, 2) GPs were provided with a state-of-the-art evidence about the effectiveness of smoking cessation care, 3) GPs’ motivation to routinely implement the guideline was identified and improved using motivational interviewing techniques, 4) GP instruction was provided and tailored to the identified implementation barriers, and 5) GPs were given the opportunity to receive additional feedback support. Previously, the effects of the multicomponent training on GPs’ provision of smoking cessation care were tested and reported elsewhere [50]. Action planning was one of the components of the GP training, and our initial RCT did not provide insight into the effects of this single behaviour change technique. Therefore, the present study focuses on a further examination of the effects and nature of action planning among the trained GPs.

### Participants

During the study period (January–August 2011), 25 GPs received a 1-h training programme that incorporated action planning. At baseline (pre-intervention), these 25 GPs saw 1,002 patients, of whom 195 (19.5%) were smokers. During post-intervention, the same GPs saw a different group of 630 patients, of whom 98 (15.6%) were smokers. In the control condition, 24 GPs and 1,769 patients (baseline: 1,066, post-intervention: 703) were included, of whom 384 (21.7%) were smoking patients (baseline: 238 (22.3%), post-intervention: 146 (20.8%)).

### Measurements

#### GP intention

Six weeks prior to the training programme, GPs rated their intention to implement guideline-recommended smoking cessation care on a 4-point scale (‘*no intention to routinely implement smoking cessation treatment within 6 months*’ (0), ‘*intention to routinely implement smoking cessation treatment within 6 months*’ (1), ‘*intention to routinely implement smoking cessation care within 1 month*’ (2), and ‘*already routinely implemented smoking cessation treatment*’ (3). To facilitate testing of the hypotheses, we used a *post-hoc* categorisation in line with the principles from the health action process approach [51] to classify GPs into three groups depending on their response to the question about their intention: 1) ‘GP pre-intenders’ (answer category 0; 4 GPs, 393 patients), ‘GP intenders’ (answer category 1 and 2 combined; 14 GPs, 2,211 patients), and ‘GP actors’ (answer category 3; 7 GPs, 797 patients).

#### Patient-reported provision of smoking cessation care

During the 3 weeks prior to and after the GP training programme, all patients completed a questionnaire immediately after their GP consultation in which they rated their GP’s smoking cessation activities during that consultation. This questionnaire included the following items: ‘*Did your GP ask you about smoking during the consultation?*’, ‘*Did your GP advise you to quit during the consultation*?’, and ‘*Did your GP refer you to any kind of follow-up quit smoking support during the consultation*’? For each item, patients could answer ‘*Yes*’ (1) or ‘*No*’ (0).

#### Action planning

During the GP training programme, action planning was assessed based on the separate plans formulated by the GP for a) identifying smokers and b) advising smokers to quit. GPs wrote down *who* was going to perform the activity, *when* the activity was going to be performed, and *how* the activity was going to be registered in the patient’s electronic health record. In addition, GPs formulated an action plan for c) arranging follow-up for smokers who are motivated to quit and a coping plan for d) arranging follow-up for smokers who are not motivated to quit. In these plans, GPs formulated the *what*, *who*, and *how* of each plan. This method is comparable to that used in similar studies with patient samples [31].

#### Specificity of GP plans

The degree of specificity of each of the components of the GPs’ plans (*who*, *when*, *what*, and *how*) was assessed using a rating method based on previous studies [31],[32],[34]. The *who component* of the plans was rated as *not completed* (0) or *completed* (1). The *when*, *what*, and *how components* of the plans were rated on a 4-point scale; components were rated as *not completed* (0) if GPs did not write down any plans, and components were rated as being *general* (1) when GPs described them in rather general terms, e.g. ‘*I will ask my patients about their smoking during the consultation*’. Components that were specified with moderate precision were rated as being *moderately specific* (2), e.g. ‘*I will ask my patients about their smoking, routinely once a year*’. A component was rated as being *highly specific* (3) when GPs specified their future action with a sufficient amount of precision, e.g. ‘*I will ask my patients about their smoking when they present with smoking-related complaints during the consultation*’.

Analyses of the *when component* showed that GPs specified either a particular moment (e.g. during the consultation), or a particular type of patient (e.g. patients with smoking-related complaints), or both; therefore, we decided to rate both these types of specifications. As a result, the total specificity score for the first two action plans (asking about smoking and advising to quit) ranged from 0 to 10, and for the third action plan (dealing with smokers who were motivated to quit) and the coping plan (dealing with smokers who were not motivated to quit), scores ranged from 0 to 7 (Appendix 1).

Two researchers independently rated the specificity of all components of the GPs’ plans. Kappa statistics were used to estimate the inter-rater agreement; this resulted in a high level of agreement between the two researchers for the total specificity scores of the GPs’ plans: i.e. for asking about smoking 0.998 (95% CI 0.995–0.999), for advising to quit 0.940 (95% CI 0.864–0.973), for arranging follow-up for smokers who are motivated to quit 0.945 (95% CI 0.850–0.978), and for arranging follow-up for smokers not motivated to quit 0.962 (95% CI 0.907–0.984). These high kappa coefficients are probably due to the type of rating method used. Disagreements were discussed until consensus was achieved. For analyses, the GPs’ total plan specificity scores were categorised into low (1) and high (2) scores, using the mean score as a cut-off.

#### Enactment of GP plans

After the GP training, we were interested in providing the GPs in the intervention group with their self-formulated if-then plans and ask them if they had the opportunity to enact them. Therefore, 6 weeks after the GP training programme, via a postal questionnaire, the GPs were asked to report the extent of plan enactment (response rate 76%; *n* = 19). In this questionnaire, each GP was provided with the four plans that they had previously formulated. GPs were asked to rate the extent to which they had enacted each plan using a 5-point scale: ‘*plan not enacted, not intending to enact in the future*’ (0), ‘*plan not enacted, intending to enact within 1 month*’(1), ‘*plan not enacted, intending to enact within a week*’ (2), ‘*plan partly enacted*’ (3),‘*plan fully enacted*’ (4). For missing data, a negative scenario was applied which assumed that GPs who did not complete the questionnaire did not enact their plans (score 0). For the analyses, scores for plan enactment were categorised into low (1) and high (2) scores using the mean score as a cut-off.

### Statistical analysis

Descriptive statistics were used for the characteristics of the GPs and for scores on specificity of the GP plan and on plan enactment. To test our hypotheses, we linked GP the data with patient data and analysed these using two-level logistic regression analyses (generalised estimating equations), including the data at the GP and patient level.

In our model, data at the GP level included scores on plan specificity and plan enactment as independent variables. To examine the main effects of these variables on GPs’ provision of smoking cessation care (patient-reported), all patients were classified into three categories, i.e. patients who had a consultation with a GP who had formulated a highly specific plan/reported a high level of plan enactment (2), patients who had a consultation with a GP who had formulated a general plan/reported a low level of plan enactment (1), and patients who had a consultation with a GP within the control condition (0).

Data at the patient level included GPs’ provision of smoking cessation care, as reported by patients, as dependent variables, including being asked about smoking, being advised to quit, and being provided with quit smoking follow-up. Patient-reported smoking cessation care was included as a dichotomous variable (1 = yes, 0 = no). The model was adjusted for differences between characteristics of the patients who visited the GPs at baseline and those who consulted the GPs post-intervention (gender, cultural background, and smoking status).

Univariate analysis was used to examine the main effects of GP plan specificity and GP-reported plan enactment on their provision of smoking cessation care (as reported by patients). In addition, interaction analysis was used to examine whether or not the effects of GP plan specificity on the delivery of care depended on the extent of GP plan enactment. Finally, subgroup analyses were performed to examine whether the effects of GP plan specificity and plan enactment on delivered smoking cessation care, differed between GPs with different baseline intentions to routinely implement smoking cessation care. In all models, we included time (baseline (0)/post-intervention (1)) by group (control group (0)/low plan specificity or low plan enactment (1)/high plan specificity or high plan enactment (2)) interaction effects since we included different cohorts of patients at baseline and post-intervention.

This study was approved by the Medical Ethical Board of the Leiden University Medical Centre (P10.125).

## Results

### Sample characteristics

Of the 49 participating GPs, 28 (57.1%) were men and 38 (77.6%) had worked more than 10 years as a GP; in addition, the majority worked on average 38 h/week and had a mean age of 50 years. Most of these GPs worked in collaboration with one (*n* = 33; 67.3%) or two (*n* = 12; 24.5%) practise nurses. None of the GP characteristics were significantly different between the intervention and control condition. A detailed overview of the background characteristics of participating GPs and patients is reported elsewhere [50].

### Specificity and enactment of GP plans

Descriptive data with regard to the specificity of GPs’ plans are presented in Table 1. Most GPs completed all components of their action plans and coping plan. With regard to the ‘when’ component, most GPs described *a type of moment* for which they planned to ask about smoking or advise to quit, instead of a *type of patient* for who they planned to provide this care. Only a minority of the GPs described the *type of moment* or the *type of patient* highly specific, such as ‘*I’ll ask my patient about smoking, when I make a risk profile of the patient*’ (moment) or ‘*I’ll ask all patients with a chronic illness about smoking*’ (patient). Only a few GPs described highly specific what they planned to do when they would encounter a smoker who is motivated or unmotivated to quit, such as ‘*When I encounter a smoker who is motivated to quit, I will discuss the (dis)advantages of quitting, motivation to quit, and I will make a quit plan*’ or ‘*When I encounter a smoker who is not motivated to quit, I’ll ask the patient’s permission to discuss their smoking behaviour again during the next consultation*’. Most GPs described highly specific *how* they planned to register the activities in the electronic patient record, for example using the ‘*International Classification of Primary Care*’. Most GPs who formulated a highly specific action plan for asking patients about smoking also reported a high level of plan enactment (*n* = 6/9, 66.7%). Similar associations were found between GP plan specificity and plan enactment in the other action and coping plans. However, some GPs who formulated general plans reported a high level of plan enactment and vice versa.

**Table 1:** **Specificity and enactment of GPs’ plans to provide guideline-recommended smoking cessation care**

	GP action plans			GP coping plan
Plan specificity (score)	Ask about smoking (***n*** = 25, 100%)	Advise to quit (***n*** = 25, 100%)	Arrange follow-up motivated to quit (***n*** = 25, 100%)	Arrange follow-up unmotivated to quit (***n*** = 25, 100%)
Who, completed (1)	24 (96.0%)	24 (96.0%)	22 (88.0%)	21 (84.0%)
When (moment)*/* what				
Not completed (0)	6 (24.0%)	6 (24.0%)	2 (8.0%)	3 (12.0%)
General (1)	13 (52.0%)	14 (56.0%)	6 (24.0%)	8 (32.0%)
Medium specific (2)	3 (12.0%)	4 (16.0%)	13 (52.0%)	5 (20.0%)
Highly specific (3)	3 (12.0%)	1 (4.0%)	4 (16.0%)	9 (36.0%)
Total score, M (SD)	1.12 (0.93)	1.00 (0.76)	1.76 (0.83)	1.80 (1.08)
When (type patient)				
Not completed (0)	20 (80.0%)	20 (80.0%)	NA	NA
General (1)	0 (0.0%)	1 (4.0%)	NA	NA
Medium specific (2)	1 (4.0%)	3 (12.0%)	NA	NA
Highly specific (3)	4 (16.0%)	1 (4.0%)	NA	NA
Total score, M (SD)	0.56 (1.16)	0.40 (0.87)	NA	NA
How register				
Not completed (0)	2 (8.0%)	1 (4.0%)	4 (16.0%)	5 (20.0%)
General (1)	4 (16.0%)	5 (20.0%)	7 (28.0%)	6 (24.0%)
Medium specific (2)	2 (8.0%)	6 (24.0%)	8 (32.0%)	10 (40.0%)
Highly specific (3)	17 (68.0%)	13 (52.0%)	6 (24.0%)	4 (16.0%)
Total score, M (SD)	2.36 (1.04)	2.24 (0.93)	1.64 (1.04)	1.52 (1.01)
Total specificity score, M (SD)ª	5.00 (2.10)	4.60 (1.66)	4.28 (1.79)	4.12 (2.03)
Plan enactment (score)				
Plan not enacted, not intending to in the future (0)	10 (40.0%)	12 (48.0%)	11 (44.0%)	15 (60.0%)
Plan not enacted, intending to within 1 month (1)	2 (8.0%)	2 (8.0%)	0 (0.0%)	1 (4.0%)
Plan not enacted, intending to within a week (2)	0 (0.0%)	0 (0.0%)	0 (0.0%)	1 (4.0%)
Plan partly enacted (3)	8 (32.0%)	6 (24%)	3 (12.0%)	3 (12.0%)
Plan fully enacted (4)	5 (20.0%)	5 (20.0%)	11 (44.0%)	5 (20.0%)
Total enactment score, M (SD)^b^	1.84 (1.70)	1.60 (1.73)	2.12 (1.94)	1.28 (1.72)

*GPs* general practitioners, *M* mean, *SD* standard deviation, *NA* not applicable.

ªTotal specificity scores for action plans ‘asking about smoking’ and ‘advising to quit’ could range from 0 to 10 and for the action and coping plans ‘arranging follow-up for smokers motivated to quit’ and ‘arranging follow-up for smokers unmotivated to quit’ could range from 0 to 7.

^b^Total enactment scores could range from 0 to 4.

### Effect of GP plan specificity and enactment on provision of smoking cessation care

Tables 2 and 3 show the effects of plan specificity and plan enactment, respectively, on GPs’ provision of smoking cessation care, contrasting patients seen by GPs in the control group. With regard to GPs’ task of ‘asking about smoking’, all patients (smokers and non-smokers) were included in the analyses but classified into patients seen by a GP 1) ‘in the control condition’, 2) ‘who formulated a general action plan’, and 3) ‘who formulated a highly specific action plan’. With regard to GPs’ tasks of ‘advising to quit’ and ‘arranging follow-up’, we present the results for the subsets of patients that reported being a smoker.

**Table 2:** **Effect of GP plan specificity on the provision of smoking cessation activities (patient-reported)** ^**a**^

	Baseline		Post-intervention		Time × group OR (95% CI)
All patients (*n* = 3,401)	*n* total	% asked	*n* total	% asked	
Asked about smoking					
Highly specific GP plan	731	29.9%	437	41.0%	2.11 (1.51–2.95)**
Low specific GP plan	271	40.3%	193	42.8%	1.29 (0.82–2.03)
Control group	1,066	40.8%	703	37.1%	1
All smokers (*n* = 665)	*n* total	% advised	*n* total	% advised	
Advised to quit					
Highly specific GP plan	93	37.1%	49	53.3%	2.28 (0.81–6.40)
Low specific GP plan	102	43.3%	49	33.3%	0.62 (0.21–1.80)
Control group	229	43.8%	143	44.1%	1
Smokers motivated to quit (*n* = 214)	*n* total	% arranged	*n* total	% arranged	
Arranged for follow-up					
Highly specific GP plan	39	15.4%	20	40.0%	^b^
Low specific GP plan	21	28.6%	11	18.2%	^b^
Control group	71	18.3%	52	9.6%	1
Smokers not motivated to quit (*n* = 408)	*n* total	% arranged	*n* total	% arranged	
Arranged for follow-up					
Highly specific GP plan	39	20.5%	21	14.3%	^b^
Low specific GP plan	82	4.9%	38	7.9%	^b^
Control group	142	4.9%	86	10.5%	1

*GPs* general practitioners, *OR* odds ratio, *CI* confidence interval.

^a^Generalised estimating equations adjusted for clustering and patient characteristics.

^b^Analyses not possible due to the sparseness of data.

*p < 0.01, **p < 0.001.

**Table 3:** **Effect of GP plan enactment on the provision of smoking cessation activities (patient-reported)** ^**a**^

	Baseline		Post-intervention		Time × group OR (95% CI)
All patients (*n* = 3,401)	*n* total	% asked	*n* total	% asked	
Asked about smoking					
High GP plan enactment	459	34.6%	314	55.7%	3.04 (2.10–4.41)**
Low GP plan enactment	543	31.1%	316	27.3%	1.01 (0.68–1.49)
Control group	1,066	40.8%	703	37.1%	1
All smokers (*n* = 665)	*n* total	% advised	*n* total	% advised	
Advised to quit					
High GP plan enactment	63	57.1%	33	66.7%	0.85 (0.27–2.65)
Low GP plan enactment	132	39.4%	65	46.2%	1.52 (0.58–3.99)
Control group	229	43.8%	143	44.1%	1
Smokers motivated to quit (*n* = 214)	*n* total	% arranged	*n* total	% arranged	
Arranged for follow-up					
High GP plan enactment	35	17.1%	16	18.1%	^b^
Low GP plan enactment	25	24.0%	15	26.7%	^b^
Control group	71	18.3%	52	9.6%	1
Smokers not motivated to quit (*n* = 408)	*n* total	% arranged	*n* total	% arranged	
Arranged for follow-up					
High GP plan enactment	35	17.1%	15	13.3%	^b^
Low GP plan enactment	86	7.0%	44	9.1%	^b^
Control group	142	4.9%	86	10.5%	1

*GPs* general practitioners, *OR* odds ratio, *CI* confidence interval.

^a^Generalised estimating equations adjusted for clustering and patient characteristics.

^b^Analyses not possible due to the sparseness of data.

*p < 0.01, **p < 0.001.

After the adjustment for clustering effects and patient characteristics, we found a significant time-by-group interaction effect of action planning on GPs asking patient about smoking (Table 2); compared to the changes on GPs’ asking about smoking in the control group, patients in the intervention group who visited their GP post-intervention reported being asked about their smoking status more often than patients who visited their GP prior to action planning. We only found a significant effect for highly specific action plans (OR 2.11, 95% CI 1.51–2.95). Similarly, we only found a positive time-by-group interaction effect of high plan enactment on GPs’ asking about smoking (Table 3; OR 3.04, 95% CI 2.10–4.41). Further analyses showed that the effect of high plan enactment on GP asking about smoking differed according to the degree of specificity of the action plan (*p* < 0.001). Compared to the changes in time in the control group, patients who visited a GP who formulated a highly specific action plan and reported a high level of plan enactment post-intervention were asked more often about their smoking behaviour compared to prior to the intervention (OR 3.08, 95% CI 2.04–4.64) (Table 4).

**Table 4:** **Interaction effect of GP plan enactment and GP plan specificity on the provision of smoking cessation activities (patient-reported)** ^**a,b**^

	Baseline		Post-intervention		Time × group OR (95% CI)
Asked about smoking	***n***total	% asked	***n***total	% asked	
High PS × high PE	359	36.5%	221	57.5%	3.08 (2.04–4.64)**
Low PS × high PE	100	24.0%	93	43.0%	3.00 (1.54–5.86)*
High PS × low PE	372	21.0%	216	20.8%	1.19 (0.74–1.92)
Low PS × low PE	171	46.8%	100	37.0%	0.71 (0.40–1.26)
Control group	1,066	40.8%	703	37.1%	1

*GPs* general practitioners, *OR* odds ratio, *CI* confidence interval, *PS* plan specificity, *PE* plan enactment.

^a^Includes all patients, both smokers and non-smokers (*n* = 3,401).

^b^Generalised estimating equations adjusted for clustering and patient characteristics.

*p < 0.01, **p < 0.001.

With regard to GPs’ plans to routinely advise smokers to quit and to arrange a follow-up for smokers who are motivated or not motivated to quit, no significant main or interaction effects of GP plan specificity and plan enactment were found on the delivery of smoking cessation care, as reported by the patients (Tables 2 and 3).

### GP intention

Table 5 presents the results of the analyses of three subgroups of patients, namely patients who consulted a GP who reported at baseline to be 1) a ‘pre-intender’, 2) an ‘intender’, or 3) an ‘actor’ regarding the implementation of smoking cessation care. For each of these subgroups, we explored whether a more specific action plan or a higher plan enactment was associated with a significant increase in the percentage of patients reporting being asked about smoking. Consistent with our hypothesis, we found no positive main effects of GP plan specificity and GP plan enactment among those patients who visited GPs who, at baseline, had already fully implemented smoking cessation care (the ‘actors’). Analyses showed a positive significant effect of high plan specificity and high plan enactment among those patients who consulted a ‘pre-intender’ GP (Table 5). Among the patients who consulted an ‘intender’ GP, both high and low plan specificities, as well as high plan enactment, had a positive effect on asking about smoking. In all the three patient subgroups, we found evidence for the combined effect of high plan specificity and high plan enactment on GP asking about smoking.

**Table 5:** **Effect of specificity and enactment of GPs’ plan on asking about smoking (patient-reported) among the three subgroups of patients who consulted 1) a pre-intender GP, 2) an intender GP, and 3) an actor GP** ^**a,b**^

	GP pre-intender (***n*** = 393)			GP intender (***n*** = 2,211)			GP actor (***n*** = 797)		
	Pre***n***total (% asked)	Post***n***total (% asked)	Time × group OR (95% CI)	Pre***n***total (% asked)	Post***n***total (% asked)	Time × group OR (95% CI)	Pre***n***total (% asked)	Post***n***total (% asked)	Time × group OR (95% CI)
Plan specificity									
High	86 (20.9%)	32 (68.8%)	8.26 (2.26–27.39)*	416 (31.0%)	274 (44.9%)	1.93 (1.49–2.50)**	229 (27.1%)	131 (20.6%)	0.82 (0.48–1.40)
Low	9 (33.3%)	0 (00.0%)	^c^	163 (33.1%)	144 (47.9%)	2.03 (1.38–2.99)**	99 (47.5%)	49 (16.3%)	0.19 (0.08–0.46)**
Control group	182 (15.4%)	84 (10.7%)	1	719 (40.1%)	495 (40.8%)	1	165 (40.6%)	124 (32.3%)	1
Plan enactment									
High	49 (28.6%)	21 (90.5%)	46.84 (6.8–324.9)**	256 (35.9%)	235 (57.0%)	2.80 (2.02–3.89)**	154 (64.3%)	58 (72.4%)	0.69 (0.36–1.32)
Low	46 (15.2%)	11 (27.3%)	1.49 (0.35–6.38)	323 (65.0%)	183 (61.2%)	1.10 (0.77–1.58)	174 (59.2%)	122 (80.3%)	0.43 (0.25–0.74)*
Control group	182 (10.0%)	84 (10.7%)	1	719 (51.3%)	495 (54.9%)	1	165 (55.5%)	124 (66.9%)	1
PS × PE									
High × high	40 (27.5%)	21 (90.5%)	66.45 (6.65–661.7)**	204 (38.2%)	166 (59.0%)	9.78 (3.90–24.53)**	115 (36.5%)	34 (29.4%)	37.82 (8.95–159.9)**
Low × high	9 (33.3%)	0 (0.00%)	^c^	52 (26.9%)	69 (52.2%)	4.78 (2.04–11.19)**	39 (17.9%)	24 (16.7%)	1.32 (0.31–5.58)
High × low	46 (15.2%)	11 (27.3%)	1.94 (0.32–11.77)	212 (24.1%)	108 (23.2%)	1.09 (0.58–2.03)	114 (17.5%)	97 (17.5%)	2.04 (0.83–5.02)
Low × low	0 (0.00%)	0 (0.00%)	^c^	111 (36.0%)	75 (44.0%)	1.60 (0.80–3.20)	60 (66.7%)	25 (16.0%)	0.14* (0.04–0.54)
Control group	182 (15.4%)	84 (10.7%)	1	719 (44.1%)	495 (41.1%)	1	165 (40.6%)	124 (32.3%)	1

*GPs* general practitioners, *PS* plan specificity, *PE* plan enactment, *OR* odds ratio, *CI* confidence interval.

^a^Includes all patients, both smokers and non-smokers (*n* = 3,401).

^b^Generalised estimating equations adjusted for clustering and patient characteristics.

^c^Analyses not possible due to the sparseness of data.

*p < 0.01, **p < 0.001.

## Discussion

### Main findings

This study examined the effects of action planning and coping planning within a training programme for GPs on their provision of guideline-recommended smoking cessation care. In line with our previously reported effects of the GP training [50], the 25 GPs in the intervention group asked patients more often about smoking after formulating an action plan during the training compared to prior to the training. In line with our hypothesis, GPs who formulated a highly specific action plan asked their patients more often about smoking than GPs with less specific plans. Moreover, high plan specificity had a positive effect on GPs asking patients about smoking when they also highly enacted their plan. The effects of plan specificity and plan enactment were particularly present among GPs who initially intended to implement smoking cessation care but who had not yet routinely implemented such care. No effects of action planning, plan specificity, and plan enactment were found on GPs’ provision of quit smoking advice and arranging follow-up for smokers who were motivated to quit. In addition, no effects were found of GP coping planning on arranging follow-up for smokers who were not motivated to quit.

### Interpretation of the findings

Our finding that action planning incorporated in a training programme for GPs increased the extent to which these professionals asked their patients about smoking is in line with the earlier results on the positive effects of incorporating self-formulated conditional plans in an educational class for health-care professionals [35]. However, no evidence was found for GP action planning on GPs’ provision of other tasks, such as advising to quit and arranging follow-up for smokers who were motivated to quit. This latter finding does not correspond with the general evidence for action planning on intended behaviours in patient samples [41]-[43]. Nevertheless, the percentage of smokers that was advised to quit smoking by GPs who formulated a highly specific-related post-intervention action plan was substantially larger compared to baseline (37.1% versus 53.3%). A comparable pattern was observed with regard to the percentage of smokers who were motivated to quit and for who a follow-up was arranged by the GP (15.4% versus 40.0%). These substantial positive changes in time were not observed within the control group (advised to quit at baseline: 43.8% versus 44.1% post-intervention; arranged follow-up for smokers motivated to quit at baseline: 18.3% versus 9.6% post-intervention).

The small sample sizes may have impeded statistical confirmation of these findings. Another explanation for this may be that GPs might have more difficulty to act upon other action plans compared to merely asking their patients about smoking. The percentage of smokers who report being advised to quit or for who follow-up support was arranged, is indeed overall lower than the percentage of patients who were asked about their smoking behaviour. Smokers tend to express more resistance and negative statements about quitting when being advised to quit compared to being asked about their smoking behaviour [52],[53]. In addition, GPs indicate that they lack an overview of health promotion programmes in their own neighbourhood to which they can refer their patients [5]. Therefore, GPs may derive more benefit from training in coping plans on how to deal with these difficulties. A second explanation might relate to the quality of the action plans, which has shown considerable variability in patient samples [17]. In the present study, although we rated the specificity of GPs’ action plans, a specific plan does not necessarily mean a ‘good’ plan. Indeed, for maximal impact of a plan, GPs require the opportunity to enact the plan as often as possible. Other aspects of planning, such as opportunity, could be explored in future studies. A final explanation may be related to the lack of a prior power analysis, which could have described the power required to detect the intended effects.

Although coping planning anticipates potential barriers to behaviour (i.e. encountering smokers who are not motivated to quit), no effect of GPs’ coping plan was found on their provision of guideline-recommended smoking cessation care to these smokers. The current guideline for smoking cessation care offers GPs a solution for this type of barrier, i.e. asking the smoker’s permission to discuss their smoking behaviour during a subsequent consultation [1]. Of our 25 GPs, only six (24%) formulated this guideline-recommended activity highly specific; this might indicate that not all GPs were familiar with this guideline-recommended solution, or that this solution may not be appropriate for all GPs. Additionally, GPs may face more specific obstacles, such as the resistance of smokers or lack of time to provide adequate smoking cessation care. Therefore, we recommend that future studies involve GPs in formulating their own obstacles and solutions to provide smoking cessation care. A volitional help sheet (providing a list of possible obstacles and behavioural responses) is often effective in translating individuals’ intention into action and might also be a suitable tool for health-care professionals [45]-[48].

We also examined the effects of plan specificity and self-reported plan enactment on GPs’ provision of smoking cessation care. In line with previous studies within patient samples, we found evidence for the positive effects of formulating a highly specific action plan on GPs’ asking about smoking compared to a general action plan [31],[51]. We also found evidence for GP-reported high plan enactment on the frequency with which GPs asked their patients about smoking. This latter finding is in line with de Vries et al. [33] and Ziegelmann et al. [34] who found that a self-reported plan enactment predicted smoking abstinence and an increase in physical activity, respectively. Moreover, our analyses showed that GPs were most likely to ask their patients about smoking when they enacted a highly specific formulated action plan. To our knowledge, this interaction effect has not yet been examined, and it provides additional insight into the mechanisms underlying action planning.

All the described effects were present among GPs who, at baseline, intended to implement smoking cessation care and were lacking among GPs who, at baseline, were already categorised as ‘actors’. These findings are in line with the theories suggesting that action planning is a post-intentional strategy which aims to bridge the gap between the individual’s intention to perform the behaviour and the actual performance of that behaviour [54],[55]. At baseline, GPs who indicated that they had already fully implemented smoking cessation care in their practise may already have a clear idea of when, where, and how they will ask their patients about smoking. Indeed, highly conscientious individuals might benefit less from self-formulated conditional plans as they may already use such approaches [17]. As reported elsewhere, the GP training programme focused on increasing the GP’s intention to implement smoking cessation care and succeeded therein [50]. This might explain why ‘pre-intender’ GPs also benefitted from action planning; however, the small size of this subgroup resulted in ORs with a wide confidence interval, indicating a low level of precision of this finding.

### Study strengths and weaknesses

The strength of the present study is that it explored whether a training programme with action planning (a strategy proven effective in patient samples) increases the provision of guideline-recommended smoking cessation activities among GPs. In addition, we examined the specificity of the plans GPs made and the extent to which they enacted these plans; these aspects are often neglected within planning interventions [17]. In particular, we distinguished between GP-reported behaviour (plan enactment) and patient-reported outcomes (GP provision of smoking cessation care). There is an increasing interest in the effects of planning interventions on the clinical behaviour of health-care professionals [56]. The present study provides further insight into the feasibility of applying this strategy in a GP sample and generates new hypotheses that can be examined in future research.

Some limitations should also be mentioned. First, we assessed the effects of the GP training incorporating action planning on patient-reported smoking cessation activities of GPs. Patients may have perceived the GP’s quit smoking advice or referral for follow-up support as being embedded in a general conversation about smoking behaviour; in that case, the smoking cessation activities of the GP might have escaped their attention. Such recall bias may have led to a lack of effect of action planning on the delivery of these smoking cessation activities. Secondly, the precise response rate of patients who completed the questionnaire (at baseline and post-intervention) is unknown. Reasons for non-response might be attributed to GPs who failed to hand out the patient questionnaires or to patients who forgot or were unwilling to complete the questionnaire. Thirdly, the relatively small sample of GPs and smoking patients might have reduced the chance of detecting a true effect of action planning, plan speci58ficity, and/or plan enactment on GPs’ provision of quit smoking advice and referrals. Also, we measured GPs’ intention and plan enactment with single item measures. Further research is needed to examine the validity of these measures. Finally, during the study period, some of the GPs did not have direct access to the smoking cessation programmes of (trained) practise nurses, which may have contributed to the lack of effect on GPs’ referrals.

## Conclusions

Action planning within a training programme for GPs improves the frequency with which the GPs ask patients about their smoking. Action planning was particularly beneficial among those GPs who had a pre-existing intention to implement smoking cessation care. Importantly, a highly specific action plan that was well enacted was most likely to result in patients being asked about smoking by their GP. Since action planning did not improve the provision of other GP tasks regarding smoking cessation care, future studies should further examine the effects of coping plans on the provision of these GP tasks. These plans might help GPs to anticipate possible barriers that impede them from acting on their intentions. In addition, we recommend that our findings be replicated in randomised controlled studies with a larger GP sample and a long-term follow-up [57].
